# Symbiotic Modulation as a Driver of Niche Expansion of Coastal Plants in the San Juan Archipelago of Washington State

**DOI:** 10.3389/fmicb.2022.868081

**Published:** 2022-06-23

**Authors:** Regina S. Redman, Joe A. Anderson, Taylor M. Biaggi, Katie E. L. Malmberg, Melissa N. Rienstra, Jamie L. Weaver, Rusty J. Rodriguez

**Affiliations:** Adaptive Symbiotic Technologies, Seattle, WA, United States

**Keywords:** fungal endophytes, stress tolerance, symbiosis, plant-fungal interactions, Class 2 endophytes

## Abstract

Modern evolutionary theory and population genetics posit that adaptation and habitat expansion of plants result from processes exclusive to their genomes. Here, we present studies showing that plants can grow across complex habitat gradients by modulating symbiotic associations with Class 2 fungal endophytes. Endophyte analysis of three native (*Leymus mollis, Distichlis spicata, and Salicornia pacifica*) and one invasive (*Spartina anglica*) plant growing across adjacent microhabitats in the San Juan Archipelago altered associations with Class 2 fungal endophytes in response to soil salinity levels. At the microhabitat interfaces where the gradation of salinity varied, the plants were colonized by endophytes from both microhabitats. A reciprocal transplant study along a salt gradient demonstrated that *Leymus mollis* (dunegrass) required endophytes indigenous to each microhabitat for optimal fitness and/or survival. In contrast, when dunegrass and *Grindelia integrifolia* (gumweed) were found growing in low salinity, but high drought habitats, these plant species had their own unique dominant endophyte association regardless of geographic proximity and conferred drought but not high salt stress tolerance. Modulation of endophyte abundance occurred *in planta* based on the ability of the symbiont to confer tolerance to the stress imposed on plants. The ability of an endophyte to confer appropriate stress tolerance resulted in a significant increase of *in planta* fungal abundance. Conversely, the inability of an endophyte to confer stress tolerance resulted in a decrease of *in planta* fungal abundance. Our studies indicate that Class 2 fungal endophytes can provide a symbiotic mechanism for niche expansion and phenotypic plasticity across environmental gradients.

## Introduction

The geographic pattern and distribution of plants across complex habitats have been extensively studied and well-documented (Martyn, 1729; Bradshaw, 1965; Crisci, 2001). The cellular and mechanistic processes responsible for the adaptive potential and phenotypic plasticity of plants are still largely undefined but thought to involve processes exclusive to the plant's genome and considered as the primary factor responsible for plant distribution patterns and biogeography (Chevin et al., 2010; Matesanz et al., 2010; Nicotra et al., 2010; Zhang et al., 2013; Gratani, 2014; Zhou et al., 2019; Liu et al., 2020; Monforte, 2020; Abady et al., 2021; Klupczyńska and Ratajczak, 2021; Stotz et al., 2021; Syngelaki et al., 2021; Yang et al., 2021; Yu et al., 2021; Wang et al., 2022). For example, some plants are adapted to the presence of selenium enabling them to grow in soils with high concentrations of the element that limit the distribution of the plant's competitors (El Mehdawi et al., 2011a,b). The biochemical basis for this adaptation is thought to involve particular proteins involved in sulfur and selenium uptake and transport, coded by genes such as SHST1, SHST2, and SHST3 (Terry et al., 2000). Since these genes are found in the plant's nuclear genome, their expression is presumed to be responsible for the adaptation. However, the first report of habitat-adapted symbiosis 20 years ago revealed that plant adaptation in high-stress habitats (Redman et al., 2002b) can occur on an intergenomic level via symbiosis with Class 2 fungal endophytes (Rodriguez et al., 2009; Goh et al., 2013). Class 2 endophytes can be transmitted vertically or horizontally, confer habitat-specific stress tolerances, can grow asymptomatically throughout the plant vegetative tissue from roots to leaves and seed coats, but not the embryo seed, and can have specific and profound effects on plant physiology (Redman et al., 2002a). For example, plants growing in geothermal soils are symbiotic with fungal endophytes that adapt the plants to heat stress but the same endophytic species growing in plants from temperate soils do not adapt plants to heat stress (Rodriguez et al., 2008). Studies have also demonstrated that without the appropriate fungal endophyte, plants are unable to compete and/or survive in stressful habitats to which they appear adapted, irrespective of the stress (Miglia et al., 2007; Kim et al., 2008; Rodriguez et al., 2008).

Over the last several decades, there have been numerous studies assessing the potential of fungal endophytes for conferring stress tolerance to plants (Redman et al., 2001; Hamilton and Bauerle, 2012; Ravindran et al., 2012; Azad and Kaminskyj, 2015; Dastogeer, 2018; Giauque et al., 2019; González-Teuber et al., 2019; Gonzalez Mateu et al., 2020; Kaur, 2020; Morsy et al., 2020). It is now clear that the ecology and adaptive potential of plants is driven, at least in part, by microbial symbionts. This is best represented by the symbiotic dynamics observed in plants growing across environmental gradients (Maciá-Vicente et al., 2012; Ranelli et al., 2015; Glynou et al., 2016; Hammami et al., 2016; Kia et al., 2018).

In plant ecology, the mechanisms responsible for the ability of plants to grow across microhabitats imposing different abiotic stresses (niche expansion) have yet to be elucidated. We hypothesized that niche expansion required either that plants associate with: (1) individual fungal endophytes that adapt to edaphic differences between adjacent microhabitats and confer appropriate stress tolerances, or (2) different endophytes specific to each microhabitat. To test these hypotheses, we assessed the symbiotic dynamics and ecological significance of Class 2 endophytes in native plant species growing across salinity gradients and in uniform low salt habitats on two islands in the San Juan Archipelago of Washington State, USA. Here, we describe a newly observed ecological phenomenon that provides a symbiotic mechanism for niche expansion and distribution of plants across complex landscapes.

## Materials and Methods

### Habitat Locations and Descriptions

**Figure 1:**
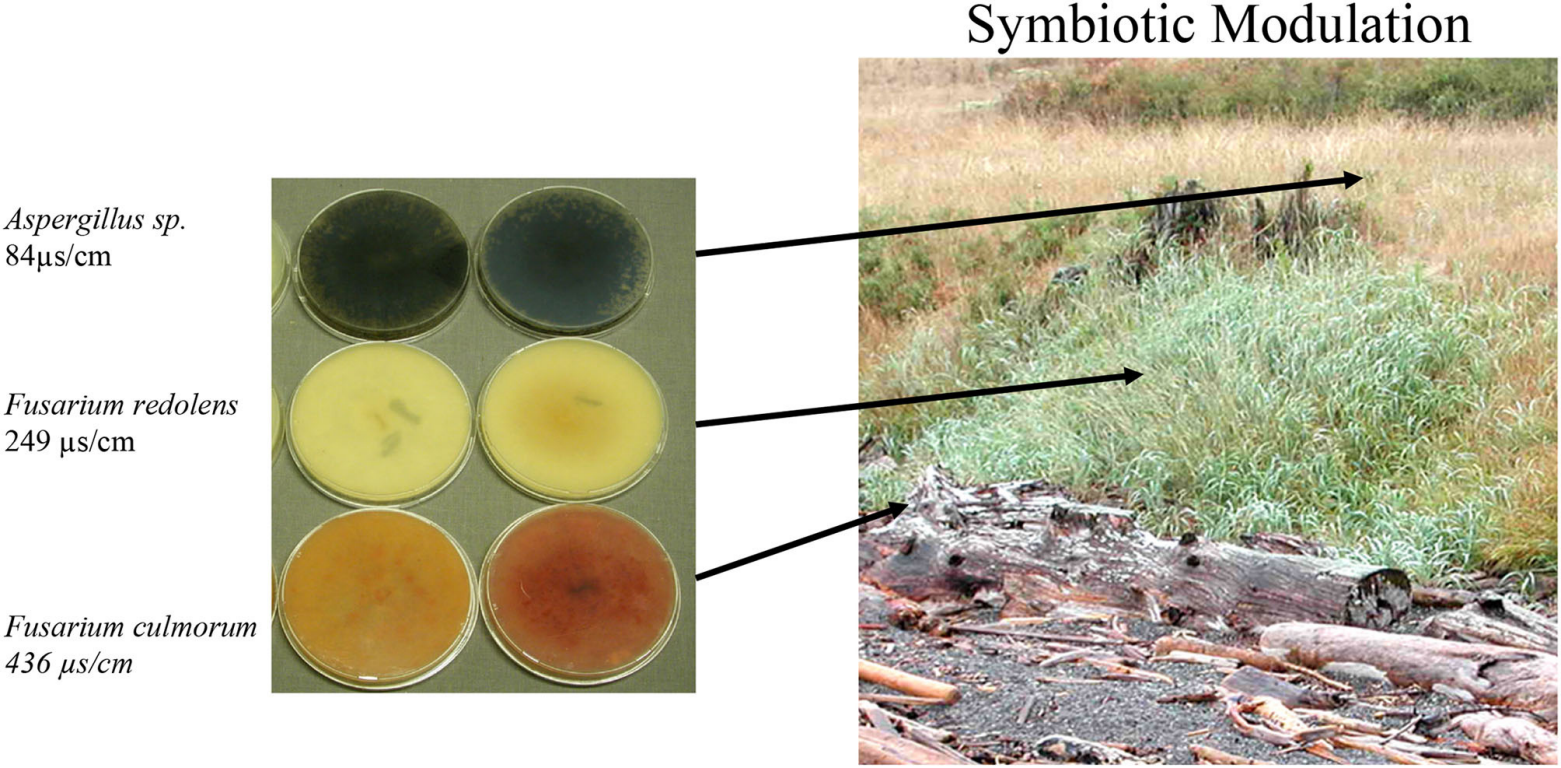
Endophyte distribution across a salt gradient. Endophytes were isolated from plants (*N* = 30/location) collected from beach, slope, and upper meadow habitats within a contiguous population of *Leymus mollis* (dunegrass). Fungal growth media plates (0.1x PDA) on the left represent the dominant endophyte (<70%) identified from plants in each location. Soil conductivity measurements were taken and the highest salinity levels found to occur on the beach, moderate levels on the slope, and low salinity levels in the upper meadow.

Five native and one invasive (*Spartina anglica*) coastal plant were collected from several locations of Shaw, Waldron, and Camano islands in the San Juan Island Archipelago, WA. Plant species were found growing along costal beach [*Salicornia pacifica* (pickleweed), *Leymus mollis* (dunegrass), *Distichlis spicata* (saltgrass), *Spartina anglica* (spartina)] and rocky cliff [*Grindelia integrifolia* (gumweed)] habitats exposed to either low or high salinity and drought conditions during summer months. Beach substrate ranged from dirt and sand to cobble and salt gradients driven by inundation from high tides and salt spray. Prior to plant sampling, the relative salinity levels surrounding the roots of plants were measured and recorded using a Stevens HydraProbe (USA) conductivity meter to delineate salt gradients. The experimental field site was located at the University of Washington's Cedar Rocks Biological Preserve on Shaw Island. At one location, dune grass had colonized beach cobble and grew up a slope (10 m) and began colonizing an upper grassland meadow (Figure 1). Slope and meadow substrate consisted of loamy soil, and the beach was cobble ranging in size from 1 cm to 3 cm.

### Endophyte Isolation

The plant species analyzed in this study and collection locations are listed in Table 1. Plants were washed to remove soil debris and any dead or decaying pant matter, placed into plastic ziplock bags, and surface-sterilized as previously described (Redman et al., 2001, 2002a). Using aseptic technique, roots, rhizomes, stems, and seed coats were separated, plated on fungal growth media (see below), and incubated at ambient temperature for 10–14 days under cool fluorescent lights until fungi emerged. Dominant endophytes were defined based on the number of fungi that emerged from surface-sterilized plant tissue sections. Endophytes found in >70% of plant samples and represented 70% of all fungi isolated/plant species/microhabitat were classified as the dominant endophytes (Figure 1, Tables 1–**4**). Ten representative isolates of the dominant fungal endophytes from each plant species were sub-cultured and single spore derived cultures generated (Redman et al., 1999). Agar plugs of mycelia from each culture were placed under sterile water supplemented with 50–100 ug/ml of ampicillin in sterile 1.5-ml screw-cap tubes and stored at 4°C for long-term storage. Taxonomic identification of the isolates is described below. The effectiveness of surface sterilization was verified using the imprint technique (Schulz et al., 1999).

**Table 1:** Dominant endophytes from plants growing across salinity gradients.

**Plant species and location**	Soil conductivity*	Endophyte morphotype**	Salt stress category***	Endophyte identity****
**Pickleweed (Salicornia pacifica), Picnic Cove, Shaw Island**				
Low Tide Zone	2,750 uS/cm	A	Extreme	*Phoma* sp.
Mid Tide Zone	1,282 uS/cm	A/B	High	*Phoma* sp./*F. culmorum*
High Tide Zone	701 uS/cm	B	Moderate	*F. culmorum*
**Dunegrass (** ***Leymus mollis*** **), Cedar Rocks, Shaw Island**				
Beach	436 μS/cm	B	Moderate	*F. culmorum*
Slope	249 μS/cm	C	Low	*Fusarium redolens*
Upper Meadow	84 μS/cm	D	Low	*Alternaria* sp.
**Dunegrass (** ***Leymus mollis*** **), Cowlitz Bay, Waldron Island**				
Beach	364 μS/cm	B	Moderate	*F. culmorum*
Slope	32 μS/cm	E	Low	*Fusarium* sp. *1*
Upper	43 μS/cm	F	Low	*Fusarium* sp. *2*
**Saltgrass (** ***Distichlis spicata*** **), Cowlitz Bay, Waldron Island**				
Low Tide Zone	23,70 μS/cm	A	Extreme	*Phoma* sp.
Mid Tide Zone	1,801 μS/cm	A/B	High	*Phoma* sp./*F. culmorum*
High Tide Zone	702 μS/cm	B	Moderate/High	*Fusarium culmorum*
**Spartina (** ***Spartina anglica*** **), Camano Island**				
Tidal Channel	4,000 μS/cm	A	Extreme	*Phoma* sp.
Slope	3,628 μS/cm	A/B	Extreme	*Phoma* sp./*F. culmorum*
Mud Flat	1,600 μS/cm	B	Moderate/High	*F. culmorum*

Salinity measurements were taken using a conductivity meter from soils in a central point where plants grew in abundance (^*^) and 10–30 plants were collected from each population for endophyte analysis. Endophytes were first placed into morphotype groups after microscopic analysis (^**^). Deionized water equates to 1.0 μS/cm, rainwater average is 50–100 μS/cm, and seawater is approximately 50,000 μS/cm. Salt stress habitats were categorized as extreme at conductivity reads <2,500 μS/cm, high at 700–2,499 μS/cm, moderate at 200–699 μS/cm, and low at a range of 0–199 μS/cm (^***^). Isolates were then further identified using DNA sequence analysis (^****^).

### Fungal Cultures

Fungal species were cultured on 0.1x potato dextrose agar (PDA) medium, supplemented with 50–100 ug/ml of ampicillin, tetracycline, and streptomycin, and grown at 22–25°C with a 12-hr cool fluorescent light regime. After 5–14 days of growth, conidia were harvested from plates by adding 10 ml of sterile water, gently scraping off spores with a sterile glass slide, and filtered through four layers of sterile cotton cheesecloth gauze. Spore concentrations were adjusted to 10^2^-10^5^ spores/ml with sterile water.

### Fungal Identification

The 10 single-spored isolates representing each dominant endophyte were identified using conidiophore and conidial morphology (Arx, 1981; Barnett and Hunter, 1998; Leslie and Summerell, 2005). DNA was isolated from three isolates of each morphotype for sequence analysis of the variable ITS1 and ITS2 sequences of rDNA [ITS4 (5'-tcctccgcttattgatatgc-3')/ITS5 (5'-ggaagtaaaagtcgtaacaagg-3') primers] and translation elongation factor [EF1T (5'-atgggtaaggaggacaagac-3')/EF2T (5'-ggaagtaccagtgatcatgtt-3') primers] (White et al., 1990; O'Donnell et al., 2000). DNA was extracted from mycelia and PCR amplified as previously described (Rodriguez and Yoder, 1991; Rodriguez, 1993). Purification and sequencing of PCR products were performed at the High-Throughput Genomics Unit, Department of Genome Sciences, University of Washington. Sequences were compared using Sequencher and BLAST searched against the GenBank database. Although genus designations were possible for all endophytes analyzed, species designations could not be made for all isolates.

### Plant Colonization

Seeds of *L. mollis* (dunegrass) obtained from their native habitats were surface-sterilized in 0.5–1.0% (v/v) sodium hypochlorite for 15–20 min with moderate agitation and rinsed with 10–20 volumes of sterile distilled water and seeds allowed to air-dry under sterile conditions. Dunegrass seeds were germinated on 1% agar medium supplemented with 1x Hoagland's solution or 0.1x PDA medium and maintained at 22–25°C and exposed to a 12-hr fluorescent light regime. To ensure that our studies began only with uninoculated (mock-inoculated with sterile water, no fungal endophytes) plants, seedlings that showed no outgrowth of fungi into the surrounding media were chosen for field and laboratory salt stress studies (see below).

*Lolium perenne* (perennial ryegrass) seeds commonly used in the Pacific Northwest were commercially purchased (Seed Factory NW, Puyallup, WA, USA), processed in a similar manner as described above for dunegrass (see above), and used for laboratory drought stress studies (see below). However, due to the small size of perennial ryegrass seeds and the high seeding rates required, a random selection of 100 seeds was chosen from the sterilized batch of seeds and plated onto fungal growth media in a similar manner as the dunegrass seeds (described above) to ensure seeds were uninoculated.

### Salt Stress

#### Propagation for Field Studies

Dunegrass seeds were transplanted into cell packs containing soil (Sunshine Mix #4). Plants were watered by filling the lower tray with 1x Hoagland's solution supplemented with 5 mM CaCl_2_. After 2 weeks, plants were either mock-inoculated with water (uninoculated) or inoculated with *Fusarium* or *Alternaria* species by pipetting 1 ml of spores (10^4^-10^5^/ml) at the base of the stems. Plants were grown under a 12-hr light regime at 25°C for 2 weeks and transferred to a cold frame greenhouse exposed to ambient temperature and light for 3 months prior to transplanting at field sites in early summer (May 2005) (Figure 1). A replicate set of one-month-old plants (20/treatment) were processed for endophyte analysis to ensure that control plants were free of fungi and that symbiotic plants contained the appropriate endophyte [*Fusarium culmorum, Fusarium redolens*, or *Alternaria sp*. (see Figure 1)]. Plants colonized with each of the three endophytes were transplanted into the beach, slope, and meadow habitats located at the University of Washington's Cedar Rocks Preserve (Shaw Island, WA). Three months after transplanting, plants were removed with root systems intact and transported back to the laboratory to assess plant viability and biomass (described below), and Class 2 fungal colonization (described above) (Figure 1, Table 2).

**Table 2:** Reciprocal transplant study on Cedar Rocks Preserve.

	Beach habitat**		Slope habitat**		Meadow habitat**	
Treatment*	**#Plants**	**Biomass**	**#Plants**	**Biomass**	**#Plants**	**Biomass**
Uninoculated control	7/20	10.2 ± 0.33^d^	20/20	9.2 ± 0.68^c^	11/20	11.1 ± 0.41^c^
Beach endophyte *F. culmorum*	**20/20** ^a^	**19.9** **±1.21**^**a**^	18/20	15.1 ± 072^b^	15/20	11.4 ± 0.74^c^
Slope endophyte *F. redolens*	16/20	17.0 ± 0.61^b^	**20/20**	**18.9** **±1.67**^**a**^	18/20	13.0 ± 016^b^
Meadow endophyte *Alternaria sp*.	12/20	13.3 ± 1.06^c^	18/20	15.0 ± 0.67^b^	**20/20**	**16.9** **±0.84**^**a**^

Seeds isolated from dunegrass plants growing across a salinity gradient were used to generate dunegrass plants under laboratory and greenhouse conditions that were either used as uninoculated controls or colonized with endophytes from the beach, slope, or upper meadow habitats (^*^). After 4 months of growth, 20 dunegrass plants for each treatment were transplanted into each of the habitats and allowed to establish for 3 months prior to assessment of plant survival, wet biomass, and endophyte profiles. The number of surviving plants at the end of the study/number of plants at the beginning of the study (^**^). Biomass is the mean number of wet grams/surviving plants at the end of the study ± standard deviation. Data were analyzed by Duncan's multiple range test (P < 0.0001). Means with the same superscripted letters (a,b,c,d) are not statistically different. Numbers in bold represent plants colonized with the endophyte indigenous to the specific niche.

#### Laboratory Salt Stress Studies

**Figure 2:**
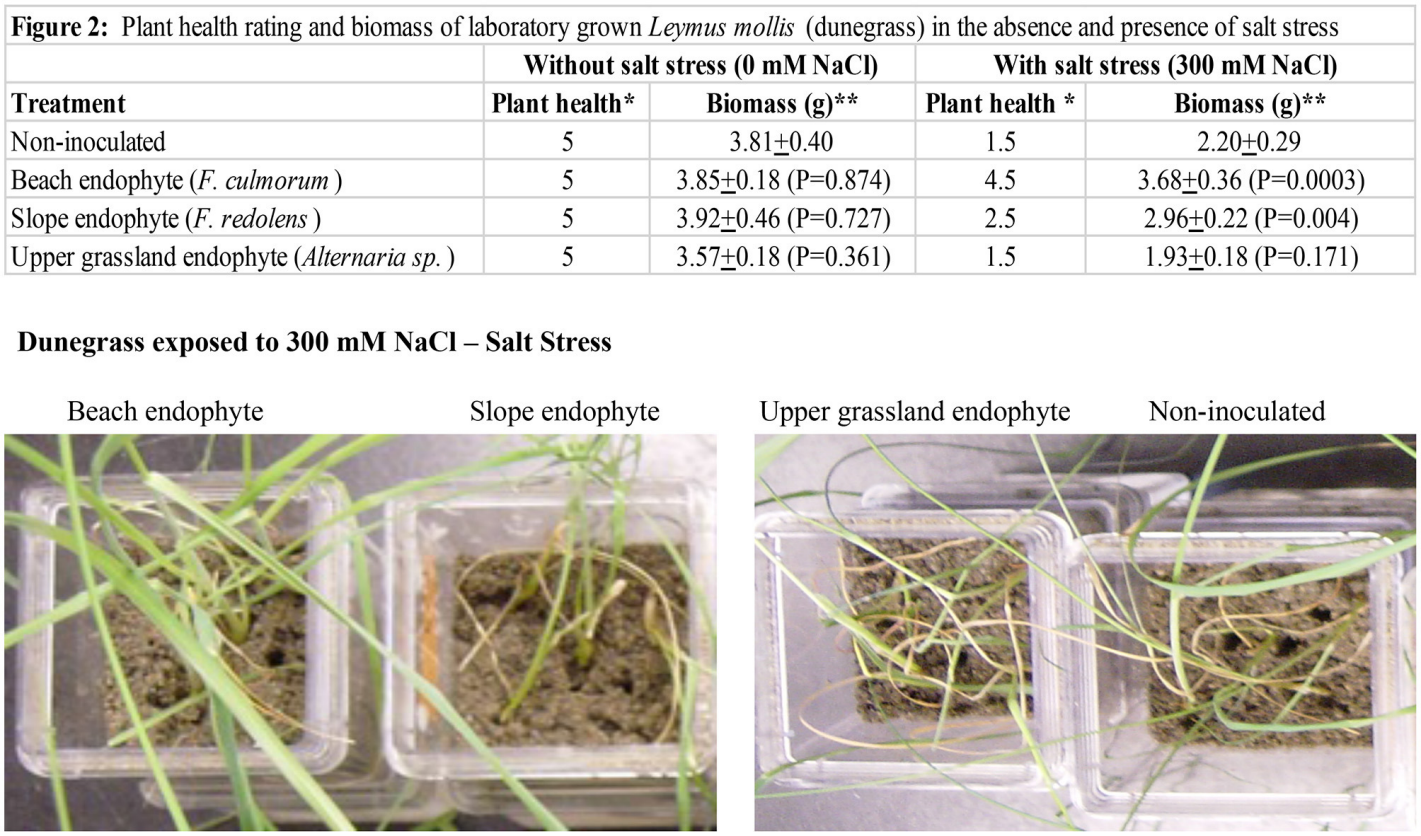
Laboratory studies were conducted in double-decker magenta boxes with four-week-old dunegrass plants (5 plants /magenta box, x10 reps of each treatment). Treatments were uninoculated controls, or symbiotically colonized *F. culmorum, F. redolens*, or *Alternaria sp*. plants. The average plant health score (*) and wet biomass (**) of five magenta boxes/treatment exposed to 0 mM or 300 mM NaCl after 2 weeks ± standard deviation values were recorded (table on top). The plant health score was assessed on a scale of 1–5 (1=dead, 2=severely wilted and chlorotic, 3=wilted +/- chlorosis, 4=slightly wilted, and 5=healthy w/o lesions or wilting). The highest salt tolerance was found with *F. culmorum*, and moderate and no salt tolerance were observed with *F. redolens* and *Alternaria sp*. plants, respectively, when compared to uninoculated controls. No differences in plant health or biomass were observed in treatments in the absence of salt stress. In the presence of 300 mM NaCl stress, differences in plant health and biomass were observed in *F. culmorum* and *F. redolens* treatments, and no plant health or biomass differences were observed in *Alternaria sp*. treatments when compared to uninoculated controls. A representative photograph showing health differences in dunegrass treatments (lower panel) exposed to salt stress (300 mM NaCl). Statistical analysis was performed using Student's *t*-test. *P* < 0.05 values were determined to be significant.

**Figure 3:**
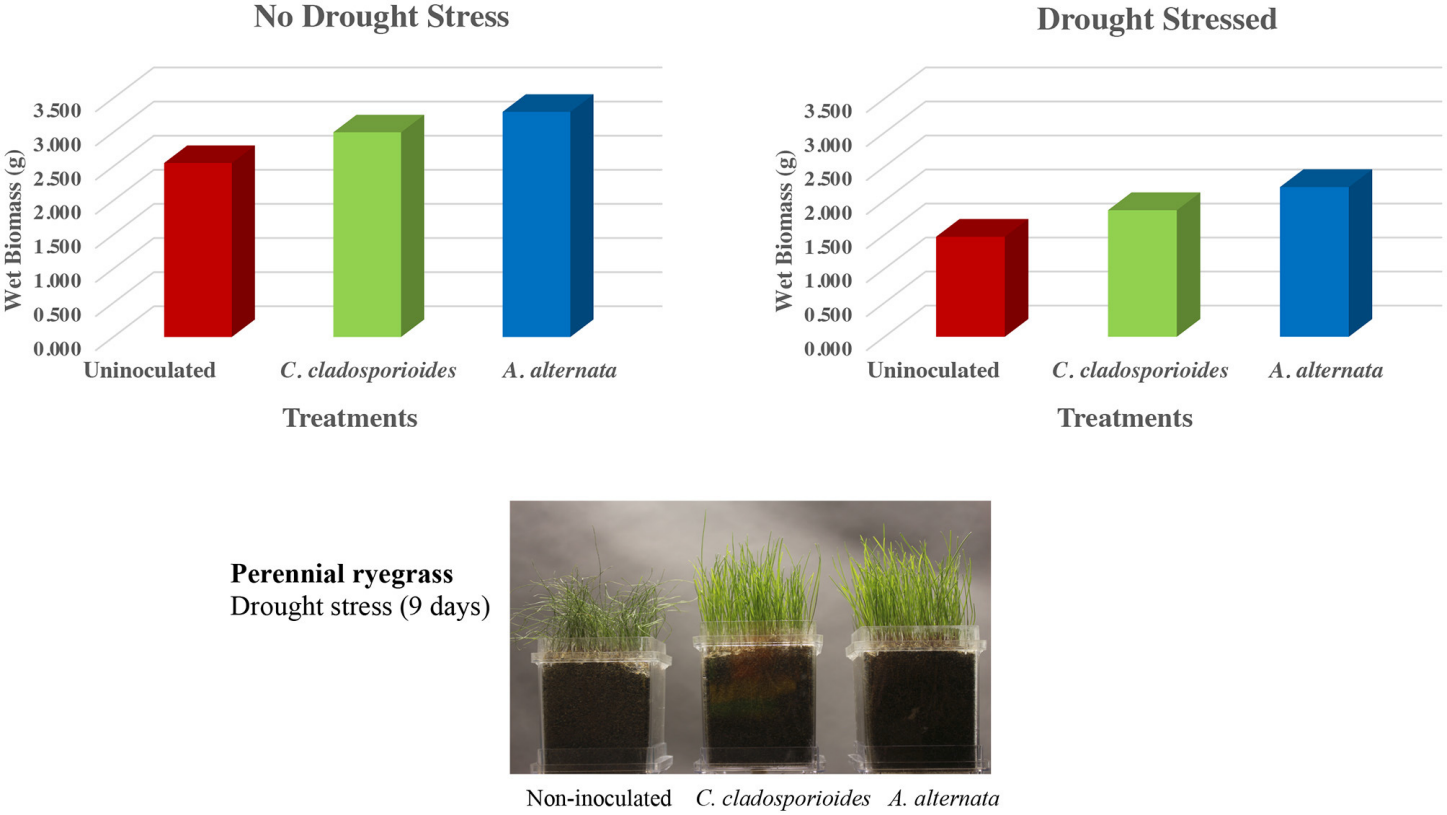
Drought stress studies in double-decker magenta boxes containing uninoculated or symbiotic (*C. cladosporioides* or *A. alternata*) treated ryegrass seeds (545 seeds/box) grown for 12 days when fluid was removed from the lower chamber of (*N* = 4 magenta boxes) to begin drought stress. Four additional magenta boxes remained as hydrated controls. After 9 days in the absence of fluids, 200 mls of fluid was added back to all the boxes and plants were allowed to recover for 7 days and the wet biomass (g) of ryegrass shoots were assessed. No significant differences in biomass were observed in the absence of stress (SD = 0.39–0.79; P = 0.209–0.394) and statistically significant differences were observed in both symbiotic plants (indicated with *; SD = 0.12–0.28; *P* = 0.014–0.016) when compared to uninoculated plants in the presence of drought stress. A representative photograph of ryegrass plants prior to shoot harvests after 9 days of drought stress (bottom panel). Statistical analysis was conducted with Student's *t*-test to determine significance between two groups (i.e., symbiotically treated vs. uninoculated controls). Values of *P* < 0.05 were considered to be statistically significant.

Experiments were performed with dunegrass plants grown in double-decker magenta boxes (Rodriguez et al., 2008) containing sand as the growth matrix and kept at 25–28°C with a 16-hr fluorescent light regime (Figure 2). Magenta boxes were randomly placed in different locations on shelves, and each experiment was repeated three times. Magenta boxes contained five dunegrass plants, and there were five replications for each treatment. Two-week-old dunegrass plants were mock-inoculated or symbiotically colonized in the same manner as described above with the three endophytes (*F. culmorum, F. redolens, and Alternaria sp*.) from the Cedar Rocks Preserve site. One-month-old plants were exposed to either no salt (1x Hoagland's solution supplemented with 5 mM CaCl_2_) or salt stress (1x Hoagland's solution supplemented with 5 mM CaCl_2_ and 300 mM NaCl) for 2 weeks by filling the lower chamber of the double-decker magenta boxes with 200 ml of one of these solutions. After plants were showing symptoms (i.e., uninoculated plants dead or severely wilted) approximately 2 weeks later, plants were re-hydrated in sterile water devoid of NaCl for 3–4 days, plant health assessed, and plants photographed (Figure 3). Plant health was assessed on a scale of 1–5 (1 = dead, 2 = severely wilted and chlorotic, 3 = wilted ± chlorosis, 4 = slightly wilted, and 5 = healthy w/o lesions or wilting). All assays were repeated a minimum of three times.

### Drought Stress

#### Laboratory Studies

Experiments were performed with perennial ryegrass plants grown in double-decker magenta boxes using sand as the growth matrix and incubated at 25–28°C with a 16-hr fluorescent light regime. Magenta boxes were randomly placed in different locations on shelves, and each experiment was repeated three times. Seven-day-old ryegrass seeds were either mock-inoculated with water (uninoculated) or inoculated with 16 spores/seed by vortexing seeds in a sterile 50-ml tube and pipetting the appropriate volume of diluted spores onto seeds. Each magenta box was seeded with 545 turfgrass seeds (1 gram), and there were four replications for each treatment. Control plants were hydrated throughout the experiment with sterile water or 1x Hoagland's solution supplemented with 5 mM CaCl_2_. Ryegrass plants were colonized with two endophytes. Both endophytes were isolated from plants growing in low salt but high drought stress sites. The first endophyte was *Cladosporium cladosporioides* from costal beach dunegrass plants, and the second endophyte was *Alternaria alternata* from gumweed plants growing on the costal rocky cliff habitats of the Cedar Rocks Preserve site (**Table 4**). Plants in magenta boxes (x8 boxes/treatment) were allowed to grow for 12 days, and the lower chamber fluid was removed from x4 boxes/treatment to begin drought conditions for 9 days; then, 200 mls fluid was added back to the lower chambers of all treatments and plants were allowed to rehydrate for 7 days. Twenty-eight-day-old ryegrass plants were photographed, shoots were harvested, and wet biomass was measured (Figure 3). All assays were repeated a minimum of three times.

### Colony-Forming Units (CFU) to Assess in Planta Modulation

**Figure 4:**
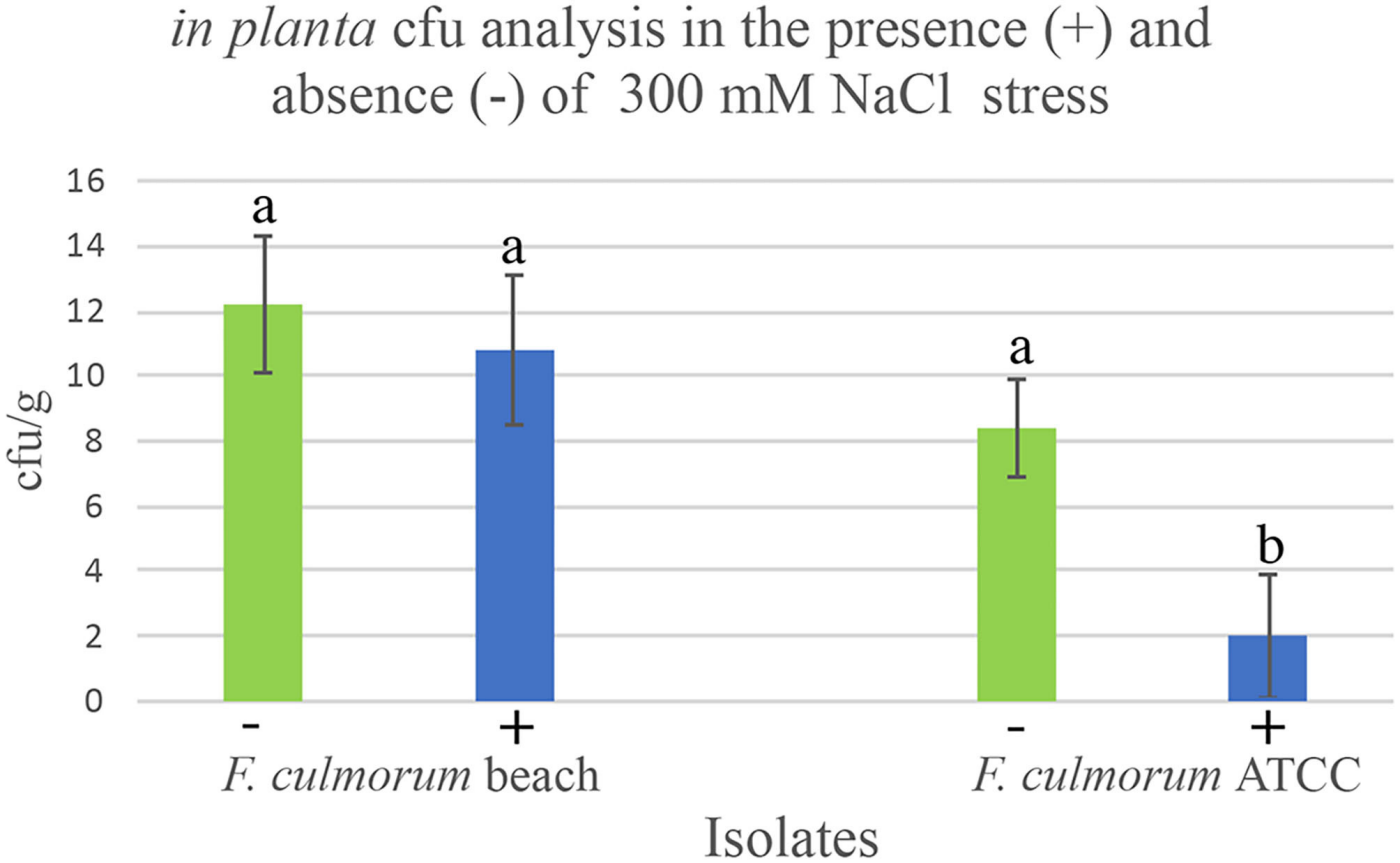
Modulation of endophytes *in planta* dunegrass (*Leymus mollis*). Laboratory studies were conducted in double-decker magenta boxes with four-week-old dunegrass plants (x5 plants/magenta box, x3 reps of each treatment) colonized with *F. culmorum* beach isolate that does impart salt tolerance or *F. culmorum* ATCC isolate that does not impart salt tolerance, exposed either to no (0 mM NaCl; green bars) or high salt stress (300 mM NaCl, blue bars) for 7 days. When visible stress symptoms were observed in the *F. culmorum* ATCC isolate, the lower stem and roots of five plants were pooled and processed for cfu analysis by grinding plant tissues in an osmotic solution and plating aliquots onto fungal growth media (0.1x PDA). The *in planta* cfu abundance of the *F. culmorum* beach isolate remained the same in the presence and absence of salt stress. In contrast, in the presence of salt stress, the abundance of *F. culmorum* ATCC isolate decreased significantly. In each graph, ± standard deviation bars are indicated and the different lowercase letters above the bars indicate significant differences (Duncan's Grouping; *P* < 0.001).

Symbiotic dunegrass plants were generated in double-decker magenta boxes in the same manner as described above. There were five dunegrass plants/magenta box and three magenta boxes per symbiotic treatments. Two-week-old plants were inoculated with *Fusarium culmorum* beach (imparts habitat-adapted salt tolerance) or *Fusarium culmorum* ATCC (from nonsalt habitat and does not impart salt stress) isolates by pipetting 1 ml of spores (10^4^/ml) at the base of the stems to generate symbiotic plants (Figure 4). One-month-old plants were exposed to 0 mM and 300 mM NaCl in 1x Hoagland's solution supplemented with 5 mM CaCl_2_ by filling the lower chamber of the double-decker magenta boxes with 200 ml with these solutions. After 7 days, when *Fusarium culmorum* ATCC symbiotic plants started showing salt stress symptoms (wilting and some chlorosis), plants were surface-sterilized (described above) and five plants were pooled with equal amounts of roots and lower stems collected and a subset of this sample equating to 0.5 g of tissue was used for the assay. Plant tissues were homogenized (Tekmar tissue homogenizer) in 10 ml of STC osmotic buffer (1M Sorbitol, 10 mM TRIS-HCl, 50 mM CaCl_2_, pH 7.5) on ice and 100 μl plated onto multiple 0.1xPDA fungal growth medium (see above). Fungal colonies (CFU) were quantified after 5–7 days and normalized to equate to CFU/g of tissue. All assays were repeated a minimum of three times.

### Statistical Analysis

Depending upon the study, *P*-values were determined by either Duncan's multiple range test, ANOVA single-factor analysis and data analyzed using SAS (SAS Institute, 2000), or Student's *t*-test.

## Results

### Coastal Plants

Four coastal plant species, growing across salt gradients on two islands of the San Juan Archipelago, changed fungal endophytes at discreet microhabitats that differed in salt concentrations determined by soil conductivity measurements (Table 1). In all plants tested, the endophytes appearing in different microhabitats represented different fungal species with typically one dominant Class 2 fungal endophyte present in>70% of plants sampled/microhabitat. None of the plants analyzed had one endophyte dominant throughout the salinity gradient. However, in microhabitat transition zones, defined by salinity levels, plants typically contained endophytes from the adjacent microhabitats (Table 1). The distribution patterns of endophytes suggested ecological function (salinity adaptation) rather than taxon-specific interactions, with endophytic species correlating to soil salinity levels, irrespective of the plant species. For example, *Fusarium culmorum* was isolated from native dunegrass (*Leymus mollis*) plants in beach habitats with similar levels of salinity irrespective of geographic location.

Dunegrass was chosen for more detailed analysis due to its importance in coastal ecosystems and distribution patterns along salt gradients (Greipsson and Davy, 1997; Houle, 2002). In one location at the University of Washington's Cedar Rocks Biological Preserve on Shaw Island, WA, dunegrass was found growing along a salinity gradient stretching 20 meters from the beach up a slope and onto an upper grassland meadow (Figure 1). Salinity levels across the gradient were 436 μS/cm (beach), 249 μS/cm (slope), and 84μS/cm (upper meadow), and one dominant endophyte was isolated from plants in each salinity microhabitat (Figure 1). Plants along the gradient differed in size and sexual reproduction. Plants growing in saline habitats were smaller, lacked plant inflorescences, and spread via, rhizomes while plants on the slope and upper meadow were large, more than twice the size of plants growing on the beach habitat. Plants growing on the slope were densely populated and robust with 70–80% inflorescences, while plants in the upper meadow grow sparsely spread out among other plant species with 40–50% of plants having inflorescences (Figure 1, Table 3). Laboratory experiments with endophytes from each microhabitat conferred levels of salt tolerance to dunegrass similar to field site salinity levels with the beach, slope, and upper meadow endophytes imparting high, moderate, and low salt tolerance, respectively, under field (Tables 2, 3) and laboratory conditions (Figure 2).

**Table 3:** Plant metrics and endophyte function across a salt gradient on Cedar Rocks Preserve.

**Microhabitat**	Endophyte ID*	Plant size**	Inflorescences**	Field site soil conductivity***
Beach	*F. culmorum*	6”-2'	0%	436 μS/cm
Slope	*F. redolens*	2'-4'	70–80%	249 μS/cm
Meadow	*Alternaria sp*.	2'-4'	40–50%	84 μS/cm

* *Endophytes were identified using microscopic and ITS sequences analysis.*

** *Plant heights and %inflorescences were determined in three random sites 3' in diameter for each microhabitat.*

*** *Conductivity of microhabitats was taken in a central point in each microhabitat*.

The ecological significance of the observed endophyte patterns was demonstrated at the Cedar Rocks site through a reciprocal transplant study of plants colonized with each dominant endophyte from each microhabitat in a factorial design where plants colonized with each of the microhabitat-specific endophytes were transplanted into each of the three microhabitats (Table 2) (Miglia et al., 2007). In every microhabitat, the highest fitness was observed by plants colonized with the endophyte indigenous to that microhabitat. Some percentage of plants colonized with nonindigenous endophytes survived in each microhabitat but achieved lower biomasses than plants with indigenous endophytes. Plant endophyte analysis revealed that during the growing season: (1) all of the plants became colonized by indigenous endophytes and (2) the indigenous endophytes became the dominant endophytes in the plants, regardless of the original colonizing endophyte or microhabitat of transplantation (Table 2). We surmise that the survival and final biomass were proportional to the timing of colonization with indigenous endophyte post-establishment.

### Endophyte Transmission

The colonization of endophyte-free plants in the transplantation study indicated soil transmission of endophytes, so it was of interest to assess potential seed transmission. Unfortunately, we could not determine seed transmission of plants in the transplant study because the site was destroyed by a violent storm. Instead, seed transmission of endophytes was assessed for two plant species: *Grindelia integrifolia* (gumweed) which commonly occurs in rocky cliff outcroppings that become very dry in summer months and *Leymus mollis* (dunegrass) which commonly occurs on the beach habitat and can be found from below the intermittent high tide line to the tops of bluffs. Endophytes were analyzed from seed coats and in roots of plants from four populations growing along a beach transect above the high tide delineation on Shaw Island. In both species, the dominant root endophytes occurred in 70–95% of all seed coats indicating very effective seed transmission (Table 4).

**Table 4:** Plant endophyte analysis in low salt (dunegrass) and high drought (gumweed) stress habitats at Cedar Rocks Preserve.

	**Plant species**	**Sampling site**	Endophyte ID*	% In plant tissues**	GPS Coordinate range***
*Leymus mollis*	x4 costal beach	*Cladosporium*	Roots/Stems	80%	48.54939 N,−122.95419 W
*Dunegrass (N = 30)*		*cladosporioides*	Seed Coat	70%	48.56431N,−122.93583 W
*Grindellia integrifolia*	x4 costal cliff	*Alternaria*	Roots/Stems	93%	48.54997 N,−122.95584 W
Gumweed (N = 42)		*alternata*	Seed Coat	95%	48.55006 N,−122.95603 W

* *Dominant endophyte analyzed by classic microbiological and DNA sequence analysis.*

** *N = 30 dunegrass plants and 576 seed coats, and N = 42 gumweed plants and 181 seed coats were analyzed for the presence of endophytes.*

*** *Plants with seed heads (approx. 70% of plants had inflorescence) were collected along a linear gradient encompassing the GPS coordinates*.

### Endophyte Modulation *In planta*

To gain insight into the mechanisms responsible for endophyte distribution patterns, we examined the relationship between *in planta* endophyte abundance in the presence and absence of salt stress of plants symbiotic with endophytes that do or do not impart salt stress tolerance. Dunegrass plants were colonized either with habitat-adapted *Fusarium culmorum* beach isolate that confers salt stress or an isolate acquired from the American type culture collection described here as *Fusarium culmorum* ATCC obtained from a habitat devoid of salt stress (Figure 4). In the absence of stress, there were no significant differences in the *in planta* colony-forming units (cfu) of endophytes recovered from plants regardless of the endophytes ability to confer stress tolerance. However, when symbiotic plants were exposed to salt stress, the fungal abundance of *F. culmorum* beach isolate remained the same while the abundance of *F. culmorum* ATCC decreased significantly. Interestingly, previous studies indicated that axenically cultured endophytes, grown on fungal growth media incubated at elevated temperatures or addition of NaCl in the medium, showed that non-habitat-adapted isolates were more tolerant of heat and salt stress *in vitro* than the habitat-adapted isolates. It appears that the stress alone does not account for the change in the *in planta* cfu (Rodriguez et al., 2008).

## Discussion

Analysis of four plant species growing across environmental gradients in different geographic locations revealed a dynamic ecological process that occurs between plants and fungal symbionts based on soil chemistry and plant fitness needs (Figure 1, Table 1). This is in line with other studies indicating that changes in soil chemistry along a chemical gradient can alter fungal endophyte associations within a single plant species (Maciá-Vicente et al., 2012; Glynou et al., 2016; Hammami et al., 2016; Kia et al., 2018). The interplay between endophyte conferred benefits and plant fitness appears to be a significant driver in plant niche expansion (Table 2). For example, the growth and development of dunegrass along the salt gradient varied significantly with regard to biomass and reproduction. None of the plants growing on the beach produced inflorescences and achieved lower biomasses than plants on the slope or upper grassland meadow (Table 3). The absence of sexual reproduction by beach plants could be viewed as decreased fitness. However, the trade-off for meiotic reproduction is salt stress tolerance and a lack of competition from other plant species (dunegrass was the solitary species in the beach cobble exposed to seawater at high tide). Moreover, dunegrass is a rhizomatous species that can grow upslope from the beach and colonize a less saline microhabitat (Figure 1). In the slope microhabitat, the plants modulate their endophytic associations to achieve optimal fitness and are able to reproduce. Although *L. mollis* (dunegrass) is known to express differential growth responses along salt gradients in costal habitats, the basis of this plasticity has not been defined (Imbert and Houle, 2000; Houle, 2002). Based on laboratory and field transplant studies, we propose that fungal endophytes are largely responsible for the plasticity of dunegrass (Figure 2, Table 2).

The interaction between imposed stress and abundance levels of endophytes *in planta* (Figure 4) suggests that endophyte composition is regulated by the host. Host regulation may explain why so few species of Class 2 endophytes are dominant in plants adapted to high-stress habitats (Table 1). Moreover, the *in planta* endophyte ecology varies between plant species which likely reflects the importance of symbiotic communication in plant biogeographical patterns. Our studies indicate that either plants communicate optimally with indigenous endophytes or exhibit decreased fitness in any specific habitat (Figure 4, Table 2).

Plant seeds can be dispersed locally or over distances into habitats to which plants are not adapted. The transmission of endophytes via seed coats ensures that newly establishing plants will emerge symbiotic but does not guarantee that the plants will be adapted for optimal fitness in distant habitats. However, as long as there are plants in the new habitats, dispersed seeds will be exposed to new fungal endophytes in the soil that can adapt germinated seeds for better fitness. This is easily envisioned along stress gradients where seed dispersal can be over very short or long distances. It is tempting to hypothesize that this type of symbiotic flexibility has allowed for the adaptive capabilities of plants to colonize complex habitats around the world.

Symbiotic associations between fungi and plants have been in existence since plants moved onto land >400 MYA and all plants are thought to be symbiotic with fungal endophytes and other microbes (Pirozynski and Malloch, 1975; Redecker et al., 2000; Krings et al., 2007). The ability of a single fungal endophyte to confer the same stress tolerance to monocots and eudicots suggests that the symbiotic communication responsible for stress tolerance is conserved and predates the divergence of these lineages (130-320 MYA (Chernikova et al., 2011; Hertweck et al., 2015). The discovery of habitat-adapted symbiosis (HAS) demonstrates that fungal endophytes can be responsible for the adaptation of plants to high-stress habitats (Rodriguez et al., 2008). The fact that Class 2 endophytes can be transmitted both vertically via seed coats and are not themselves stress-tolerant, indicates that endophyte conferred stress tolerance is an epigenetic phenomenon. Here, we demonstrate that as plants are exposed to changing environmental conditions, they modulate associations with fungal endophytes to optimize fitness. This “symbiotic modulation” is a dynamic aspect of HAS allowing individual plant species to grow as continuous populations across chemical gradients. We hypothesize that the ability of plants to modulate symbiotic associations provides a mechanistic basis for phenotypic plasticity, adaptation, niche expansion, and the biogeography of plants.

## Data Availability Statement

The datasets presented in this study can be found in online repositories. The names of the repository/repositories and accession number(s) can be found below: NCBI GeneBank - OM816650, OM816673, OM812680, OM826983, OM826984, OM826979, OM826980, and OM812800.

## Author Contributions

RSR and RJR conceived and designed the experiments, performed the experiments, contributed reagents/materials/analysis tools, and wrote the paper. RSR, JA, TB, KM, MR, and JW analyzed the data. All authors contributed to the article and approved the submitted version.

## Funding

Funding was provided by USGS, NSF (0414463), US/IS BARD (3260-01C), and ARO (54120-LS).

## Conflict of Interest

RSR, JA, TB, KM, MR, JW, and RJR were employed by Adaptive Symbiotic Technologies.

## Publisher's Note

All claims expressed in this article are solely those of the authors and do not necessarily represent those of their affiliated organizations, or those of the publisher, the editors and the reviewers. Any product that may be evaluated in this article, or claim that may be made by its manufacturer, is not guaranteed or endorsed by the publisher.

## References

[B1] Abady S. Shimelis H. Janila P. Yaduru S. Shayanowako A. I. T. Deshmukh D.. (2021). Assessment of the genetic diversity and population structure of groundnut germplasm collections using phenotypic traits and SNP markers: implications for drought tolerance breeding. PLoS ONE 16, e0259883. 10.1371/journal.pone.0259883 34788339 PMC8598071

[B2] Arx J. A. V. (1981). The Genera of Fungi Sporulating in Pure Culture, 3rd Revised Edn. Vaduz: Lubrecht and Cramer Ltd. p. 1–424.

[B3] Azad K. Kaminskyj S. (2015). A fungal endophyte strategy for mitigating the effect of salt and drought stress on plant growth. Symbiosis 68, 73–78. 10.1007/s13199-015-0370-y

[B4] Barnett H. L. Hunter B. B. (1998). Illustrated Genera of Imperfect Fungi. St. Paul, American Phytopathology Society.

[B5] Bradshaw A. D. (1965). Evolutionary significance of phenotypic plasticity in plants. Adv. Genet. 13, 115–155. 10.1016/S0065-2660(08)60048-6

[B6] Chernikova D. Motamedi S. Csürös M. Koonin E. Rogozin I. (2011). A late origin of the extant eukaryotic diversity: divergence time estimates using rare genomic changes. Biol. Direct 6, 1–18. 10.1186/1745-6150-6-26 21595937 PMC3125394

[B7] Chevin L. M. Lande R. Mace G. M. (2010). Adaptation, plasticity, and extinction in a changing environment: towards a predictive theory. PLoS Biol. 8, 1–8. 10.1371/journal.pbio.1000357 20463950 PMC2864732

[B8] Crisci J. V. (2001). The voice of historical biogeography. J. Biogeogr. 28, 157–168. 10.1046/j.1365-2699.2001.00523.x

[B9] Dastogeer K. (2018). Influence of fungal endophytes on plant physiology is more pronounced under stress than well-watered conditions: a meta-analysis. Planta 248, 1403–1416. 10.1007/s00425-018-2982-y 30121874

[B10] El Mehdawi A. F. Quinn C. F. Pilon-Smits E. A. H. (2011a). Effects of selenium hyperaccumulation on plant–plant interactions: evidence for elemental allelopathy? New Phytol. 191, 120–131. 10.1111/j.1469-8137.2011.03670.x 21371042

[B11] El Mehdawi A. F. Quinn C. F. Pilon-Smits E. A. H. (2011b). Selenium hyperaccumulators facilitate selenium-tolerant neighbors via phytoenrichment and reduced herbivory. Curr. Biol. 21, 1440–1449. 10.1016/j.cub.2011.07.033 21856156

[B12] Giauque H. Connor E. W. Hawkes C. V. (2019). Endophyte traits relevant to stress tolerance, resource use and habitat of origin predict effects on host plants. New Phytol. 221, 2239–2249. 10.1111/nph.15504 30276818

[B13] Glynou K. Ali T. Buch A. K. Haghi Kia S. Ploch S. Xia X.. (2016). The local environment determines the assembly of root endophytic fungi at a continental scale. Environ. Microbiol. 18, 2418–2434. 10.1111/1462-2920.13112 26530450

[B14] Goh C. H. Veliz Vallejos D. F. Nicotra A. B. Mathesius U. (2013). The impact of beneficial plant-associated microbes on plant phenotypic plasticity. J. Chem. Ecol. 39, 826–839. 10.1007/s10886-013-0326-8 23892542 PMC3738838

[B15] Gonzalez Mateu M. Baldwin A. H. Maul J. E. Yarwood S. A. (2020). Dark septate endophyte improves salt tolerance of native and invasive lineages of Phragmites australis. ISME J. 14, 1943–1954. 10.1038/s41396-020-0654-y 32341473 PMC7367851

[B16] González-Teuber M. Urzúa A. Morales A. Ibáñez C. Bascuñán-Godoy L. (2019). Benefits of a root fungal endophyte on physiological processes and growth of the vulnerable legume tree *Prosopis chilensis* (Fabaceae). J. Plant Ecol. 12, 264–271. 10.1093/jpe/rty019

[B17] Gratani L. (2014). Plant phenotypic plasticity in response to environmental factors. Adv. Bot. 2014, 1–17. 10.1155/2014/208747

[B18] Greipsson S. Davy A. J. (1997). Responses of *Leymus arenarius* to nutrients: improvement of seed production and seedling establishment for land reclamation. J. Appl. Ecol. 34, 1165–1176. 10.2307/2405229

[B19] Hamilton C. Bauerle T. (2012). A new currency for mutualism? Fungal endophytes alter antioxidant activity in hosts responding to drought. Fungal Divers. 54, 39–49. 10.1007/s13225-012-0156-y

[B20] Hammami H. Baptista P. Martins F. Gomes T. Abdelly C. Mahmoud O. (2016). Impact of a natural soil salinity gradient on fungal endophytes in wild barley (*Hordeum maritimum* With.). World J. Microbiol. Biotechnol. 32, 1–11. 10.1007/s11274-016-2142-0 27655527

[B21] Hertweck K. L. Kinney M. S. Stuart S. A. Maurin O. Mathews S. Chase M. W.. (2015). Phylogenetics, divergence times and diversification from three genomic partitions in monocots. Bot. J. Linn. Soc. 178, 375–393. 10.1111/boj.12260

[B22] Houle G. (2002). Trade-off between growth ability and stress tolerance in Leymus mollis (Poaceae) along a subarctic coastal dune sequence in northern Quebec. Canad. J. Bot. 80, 869–874. 10.1139/b02-073

[B23] Imbert E. Houle G. (2000). Ecophysiological differences among Leymus mollis populations across a subarctic dune system caused by environmental, not genetic, factors. New Phytol. 147, 601–608. 10.1046/j.1469-8137.2000.00724.x 33862942

[B24] Kaur T. (2020). Fungal endophyte-host plant interactions: Role in sustainable agriculture, in Sustainable Crop Production (IntechOpen). p. 1–18.

[B25] Kia S. H. Jurkechova M. Glynou K. Piepenbring M. Maciá-Vicente J. G. (2018). The effects of fungal root endophytes on plant growth are stable along gradients of abiotic habitat conditions. FEMS Microbiol. Ecol. 94:fix162. 10.1093/femsec/fix162 29186430

[B26] Kim Y. O. Rodriguez R. J. Lee E. J. Redman R. S. (2008). Phytolacca americana from contaminated and noncontaminated soils of South Korea: effects of elevated temperature, CO2 and simulated acid rain on plant growth response. J. Chem. Ecol. 34, 1501–1509. 10.1007/s10886-008-9552-x 18956232

[B27] Klupczyńska E. A. Ratajczak E. (2021). Can Forest Trees Cope with Climate Change?—Effects of DNA Methylation on Gene Expression and Adaptation to Environmental Change. Int. J. Mol. Sci. 22, 13524. 10.3390/ijms222413524 34948318 PMC8703565

[B28] Krings M. Taylor T. N. Hass H. Kerp H. Dotzler N. Hermsen E. J. (2007). Fungal endophytes in a 400-million-yr-old land plant: infection pathways, spatial distribution, host responses. New Phytol. 174, 648–657. 10.1111/j.1469-8137.2007.02008.x 17447919

[B29] Leslie J. F. Summerell B. A. (2005). The Fusarium Laboratory Manual. Ames, Blackwell Publishing.

[B30] Liu N. Du Y. Warburton M. L. Xiao Y. Yan J. (2020). Phenotypic plasticity contributes to maize adaptation and heterosis. Mol. Biol. Evol. 38, 1262–1275. 10.1093/molbev/msaa283 33212480 PMC8480182

[B31] Maciá-Vicente J. Ferraro V. Burruano S. Lopez-Llorca L. (2012). Fungal assemblages associated with roots of halophytic and non-halophytic plant species vary differentially along a salinity gradient. Microb. Ecol. 64, 668–679. 10.1007/s00248-012-0066-2 22573239

[B32] Martyn J. (1729). An account of some observations relating to natural history, made in a journey to the peak in derbyshire. Philos. Trans. 36, 22–32. 10.1098/rstl.1729.0005

[B33] Matesanz S. Gianoli E. Valladares F. (2010). Global change and the evolution of phenotypic plasticity in plants. Ann. N. Y. Acad. Sci. 1206, 35–55. 10.1111/j.1749-6632.2010.05704.x 20860682

[B34] Miglia K. J. McArthur E. D. Redman R. S. Rodriguez R. J. Zak J. C. Freeman D. C. (2007). Genotype, soil type, and locale effects on reciprocal transplant vigor, endophyte growth, and microbial functional diversity of a narrow sagebrush hybrid zone in Salt Creek, Canyon, Utah. Am. J. Bot. 94, 425–436. 10.3732/ajb.94.3.425 21636412

[B35] Monforte A. J. (2020). Time to exploit phenotypic plasticity. J. Exp. Bot. 71, 5295–5297. 10.1093/jxb/eraa268 32949243 PMC7501808

[B36] Morsy M. Cleckler B. Armuelles-Millican H. (2020). Fungal endophytes promote tomato growth and enhance drought and salt tolerance. Plants 9, 877. 10.3390/plants9070877 32664321 PMC7411952

[B37] Nicotra A. B. Atkin O. K. Bonser S. P. Davidson A. M. Finnegan E. J. Mathesius U.. (2010). Plant phenotypic plasticity in a changing climate. Trends Plant Sci. 15, 684–692. 10.1016/j.tplants.2010.09.008 20970368

[B38] O'Donnell K. Kistler H. C. Tacke B. K. Casper H. C. (2000). Gene genealogies reveal global phylogeographic structure and reproductive isolation among lineages of *Fusarium graminearum*, the fungus causing wheat scab. Proc. Natl. Acad. Sci. 97, 7905–7910. 10.1073/pnas.130193297 10869425 PMC16643

[B39] Pirozynski K. A. Malloch D. W. (1975). The origin of land plants a matter of mycotrophism. Biosystems 6, 153–164. 10.1016/0303-2647(75)90023-4 1120179

[B40] Ranelli L. B. Hendricks W. Q. Lynn J. S. Kivlin S. N. Rudgers J. A. Diez J. (2015). Biotic and abiotic predictors of fungal colonization in grasses of the Colorado Rockies. Divers Distrib. 21, 962–976. 10.1111/ddi.12310

[B41] Ravindran C. Naveenan T. Varatharajan G. R. Rajasabapathy R. Meena R. M. (2012). Antioxidants in mangrove plants and endophytic fungal associations. Botanica Marina. 55, 269–279. 10.1515/bot-2011-0095

[B42] Redecker D. Kodner R. Graham L. E. (2000). Glomalean fungi from the Ordovician. Science 289, 1920–1921. 10.1126/science.289.5486.1920 10988069

[B43] Redman R. S. Dunigan D. D. Rodriguez R. J. (2001). Fungal symbiosis: from mutualism to parasitism, who controls the outcome, host or invader? New Phytol. 151, 705–716. 10.1046/j.0028-646x.2001.00210.x 33853254

[B44] Redman R. S. Freeman S. Clifton D. R. Morrel J. Brown G. Rodriguez R. J. (1999). Biochemical analysis of plant protection afforded by a nonpathogenic endophytic mutant of colletotrichum magna. Plant Physiol. 119, 795–804. 10.1104/pp.119.2.795 9952476 PMC32157

[B45] Redman R. S. Rossinck M. R. Maher S. Andrews Q. C. Schneider W. L. Rodriguez R. J. (2002a). Field performance of cucurbit and tomato plants infected with a nonpathogenic mutant of *Colletotrichum magna* (teleomorph: *Glomerella magna*; Jenkins and Winstead). Symbiosis 32, 55–70.

[B46] Redman R. S. Sheehan K. B. Stout R. G. Rodriguez R. J. Henson J. M. (2002b). Thermotolerance conferred to plant host and fungal endophyte during mutualistic symbiosis. Science 298, 1581. 10.1126/science.1078055 12446900

[B47] Rodriguez R. J. (1993). Polyphosphates present in DNA preparations from filamentous fungal species of *Colletotrichum* inhibits restriction endonucleases and other enzymes. Anal. Biochem. 209, 1–7. 10.1006/abio.1993.1122 8385889

[B48] Rodriguez R. J. Henson J. Van Volkenburgh E. Hoy M. Wright L. Beckwith F.. (2008). Stress Tolerance in Plants via Habitat-Adapted Symbiosis. Int. Soc. Microb. Ecol 2, 404–416. 10.1038/ismej.2007.106 18256707

[B49] Rodriguez R. J. White J. F. J. Arnold A. E. Redman R. S. (2009). Fungal endophytes: diversity and functional roles. New Phytol 182, 314–330. 10.1111/j.1469-8137.2009.02773.x 19236579

[B50] Rodriguez R. J. Yoder O. C. (1991). A family of conserved repetitive DNA elements from the fungal plant pathogen Glomerella cingulata (*Colletotrichum lindemuthianum*). Exp. Mycol. 15, 232–242. 10.1016/0147-5975(91)90025-9

[B51] Schulz B. Rommert A. K. Dammann U. Aust H. J. Strack D. (1999). The endophyte-host interaction: a balanced antagonism? Mycol. Res. 10, 1275–1283. 10.1017/S0953756299008540

[B52] Stotz G. C. Salgado-Luarte C. Escobedo V. M. Valladares F. Gianoli E. Penuelas J. (2021). Global trends in phenotypic plasticity of plants. Ecol. Lett. 24, 2267–2281. 10.1111/ele.13827 34216183

[B53] Syngelaki E. Paetzold C. Hörandl E. (2021). Gene expression profiles suggest a better cold acclimation of polyploids in the alpine species ranunculus kuepferi (Ranunculaceae). Genes 12, 1818. 10.3390/genes12111818 34828424 PMC8625111

[B54] Terry N. Zayed A. M. de Souza M. P. Tarun A. S. (2000). Selenium in higher plants. Ann. Rev. Plant Physiol. Plant Mol. Biol. 51, 401–432. 10.1146/annurev.arplant.51.1.401 15012198

[B55] Wang Z. Baskin J. M. Baskin C. C. Yang X. Liu G. Ye X.. (2022). Great granny still ruling from the grave: Phenotypical response of plant performance and seed functional traits to salt stress affects multiple generations of a halophyte. J. Ecol. 110, 117–128. 10.1111/1365-2745.13789

[B56] White T. J. Bruns T. Lee S. Taylor J. (1990). Amplification and direct sequencing of fungal ribosomal RNA genes for phylogenetics, in PCR Protocols: A Guide to Methods and Applications, eds Innis M. A. Gelfand D. H. Sninsky J. J. White T. J. (San Diego: Academic Press, INC.), 315–322.

[B57] Yang Z. Bai C. Wang P. Fu W. Wang L. Song Z.. (2021). Sandbur drought tolerance reflects phenotypic plasticity based on the accumulation of sugars, lipids, and flavonoid intermediates and the scavenging of reactive oxygen species in the root. Int. J. Mol. Sci. 22, 12615. 10.3390/ijms222312615 34884421 PMC8657935

[B58] Yu H. Zhao X. Huang W. Zhan J. He Y. (2021). Drought stress influences the growth and physiological characteristics of solanum rostratum dunal seedlings from different geographical populations in China. Front. Plant Sci. 12, 733268. 10.3389/fpls.2021.733268 34868115 PMC8637895

[B59] Zhang Y. Y. Fischer M. Colot V. Bossdorf O. (2013). Epigenetic variation creates potential for evolution of plant phenotypic plasticity. New Phytol. 197, 314–322. 10.1111/nph.12010 23121242

[B60] Zhou B. Kang Y. Leng J. Xu Q. (2019). Genome-Wide Analysis of the miRNA–mRNAs Network Involved in Cold Tolerance in Populus simonii × P. nigra. Genes 10, 430. 10.3390/genes10060430 31195761 PMC6627750

